# Robust Candidates for Language Development and Evolution Are Significantly Dysregulated in the Blood of People With Williams Syndrome

**DOI:** 10.3389/fnins.2019.00258

**Published:** 2019-03-26

**Authors:** Antonio Benítez-Burraco, Ryo Kimura

**Affiliations:** ^1^Department of Spanish, Linguistics, and Theory of Literature (Linguistics), Faculty of Philology, University of Seville, Seville, Spain; ^2^Department of Anatomy and Developmental Biology, Graduate School of Medicine, Kyoto University, Kyoto, Japan

**Keywords:** Williams syndrome, blood transcriptional profile, language disorders, language evolution, candidate genes

## Abstract

Williams syndrome (WS) is a clinical condition, involving cognitive deficits and an uneven language profile, which has been the object of intense inquiry over the last decades. Although WS results from the hemideletion of around two dozen genes in chromosome 7, no gene has yet been probed to account for, or contribute significantly to, the language problems exhibited by the affected people. In this paper we have relied on gene expression profiles in the peripheral blood of WS patients obtained by microarray analysis and show that several robust candidates for language disorders and/or for language evolution in the species, all of them located outside the hemideleted region, are up- or downregulated in the blood of subjects with WS. Most of these genes play a role in the development and function of brain areas involved in language processing, which exhibit structural and functional anomalies in people with this condition. Overall, these genes emerge as robust candidates for language dysfunction in WS.

## Introduction

Williams syndrome (WS) is a clinical condition resulting from a hemizygous deletion of 1.5 to 1.8 Mb on 7q11.23, which encompasses nearly 30 genes (Korenberg et al., 2000; Pober et al., 2010). The affected people exhibit a distinctive behavioral and cognitive profile, with enhanced sociability, mental retardation, impaired spatial cognition, and spared musical abilities (Reilly et al., 1990; Udwin and Yule, 1991; Bellugi et al., 1999; Galaburda et al., 2002; Levitin et al., 2005). Language abilities are significantly preserved in people with WS compared to other neurodevelopmental disorders, to the extent that this syndrome has often been used to support the view that language can be teased apart from other aspects of cognition. Nonetheless, recent, fine-grained analyses of WS language have concluded that WS language is delayed or impaired across different levels compared to the neurotypical population (Karmiloff-Smith and Mills, 2006; Brock, 2007; Mervis and Becerra, 2007; Martens et al., 2008 for good reviews). Specifically, children with WS experience problems with irregular word forms and complex syntax; likewise, they have problems with word definitions, although they usually excel on expressive vocabulary (including semantic organization and fluency) (Volterra et al., 1996; Mervis et al., 1999; Purser et al., 2011; Van Den Heuvel et al., 2016; see Mervis and Becerra, 2007 for discussion). However, as with other aspects of the cognitive profile of this condition, no robust gene-to-phenotype associations have been established in the language domain. To date, the most promising candidates for language dysfunction in WS are *GTF2I*, *BAZ1B*, and *LIMK1*. In particular, *GTF2I*, which encodes a regulator of transcription, has been repeatedly related to the behavioral and cognitive disabilities that are typically found in this condition and that have an impact on language function (Morris et al., 2003; Tassabehji et al., 2005; Sakurai et al., 2011; Hoeft et al., 2014). Its adjacent paralog, *GTF2IRD1*, has been related to altered vocalizations among other features (Howard et al., 2012). Interestingly too, *BAZ1B* haploinsufficiency explains almost 50% of transcriptional dysregulation in WS neurons, with BAZ1B target genes being enriched in functions related to neurogenesis and neuron differentiation (Lalli et al., 2016). Regarding *LIMK1*, it regulates synaptic plasticity and long-term memory (Todorovski et al., 2015), and its hemideletion has been hypothesized to account for the observed deficits in spatial cognition in combination with other genes (Gray et al., 2006; Smith et al., 2009). Still, these potential links with aspects of language (dys)function seem quite vague, particularly if one considers our remarkable understanding of the genetic underpinnings of human language, language disorders, and language evolution (see Scharff and White, 2004; Li and Bartlett, 2012; Benítez-Burraco, 2013; Graham et al., 2015; Fisher, 2017; Murphy and Benítez-Burraco, 2017, 2018 for reviews). Examining how robust candidate genes for language disorders and language evolution behave in people with WS should help refine our view of the molecular causes of the language deficits attested in this condition. One general reason supporting this approach is the deep link that exists between evolution and (abnormal) development, in the spirit of evo-devo theories. One specific reason supporting this approach is that although in WS the number of hemideleted genes is small, changes in the dosage of hundreds, or even thousands, of other genes can be expected, with a potential impact on language abilities, in the spirit of omnigenic theories of complex diseases (Boyle et al., 2017; Peedicayil and Grayson, 2018). Recently Kimura et al. (2018) confirmed that the dysregulation of several co-expression modules involving dozens of genes outside of the 7q11.23 region seemingly accounts for the complex phenotypes observed in WS patients. Importantly, they found *BCL11A*, a gene associated with speech disorders, among the hub genes in the top WS-related modules.

In this paper we have conducted a more focused research on the potential dysregulation of genes related to language outside the WS region as a possible explanation of the distinctive language profile of the affected people. Similarly to Kimura et al. (2018), we have relied on gene expression profiles in peripheral blood of WS patients obtained by microarray analysis. We have found that significant differences exist in the blood of subjects with WS compared to neurotypical controls in the expression levels of robust candidates for language development, language evolution, and language impairment.

## Methods

The list of core candidates for language (abnormal) development and language evolution (Supplementary Table S1) encompasses two subsets of genes. The first subset consists of strong candidates for language disorders, in particular, developmental dyslexia (DD) and specific language disorder (SLI), as listed by Paracchini et al. (2016), Pettigrew et al. (2016) and Chen et al. (2017). The second subset consists of strong candidates for language evolution, as compiled by Boeckx and Benítez-Burraco (2014a,b) and Benítez-Burraco and Boeckx (2015). These are genes involved in the globularization of the human skull/brain and the cognitive changes accounting for our species-specific ability to learn and use languages (aka our *language- readiness*). Overall, the genes comprising this second subset fulfill several criteria. First, they have changed (and/or interact with genes that have changed) after our split from Neanderthals/Denisovans, including changes in their coding regions and/or their epigenetic profile. Second, they play some known role in brain development, regionalization, wiring, and/or function. Third, they are candidates for language dysfunction in broad cognitive disorders, particularly, autism spectrum disorder (ASD) and schizophrenia (SZ) (see Benítez-Burraco and Murphy, 2016; Murphy and Benítez-Burraco, 2016, 2017 for details about their role in language processing).

The gene expression profiling data of peripheral blood were obtained from our previous study (Kimura et al., 2018), available at the Gene Expression Omnibus (GSE89594). Briefly, total RNA from 32 WS patients and 30 healthy controls were analyzed using an Agilent Human GE v2 8×60K Microarray (Agilent Technologies). After the normalization step, differentially expressed genes (DEG) were calculated using the Limma R package (Smyth, 2005). The Benjamini-Hochberg method was used to evaluate the false discovery rate (FDR) (Benjamini and Hochberg, 1995). DEG were defined as FDR < 0.05 and the |fold change (FC)| > 1.2. Gene list enrichment analysis was performed using Fisher’s exact test. All the expressed genes were used as the background gene list.

## Results

**FIGURE 1:**
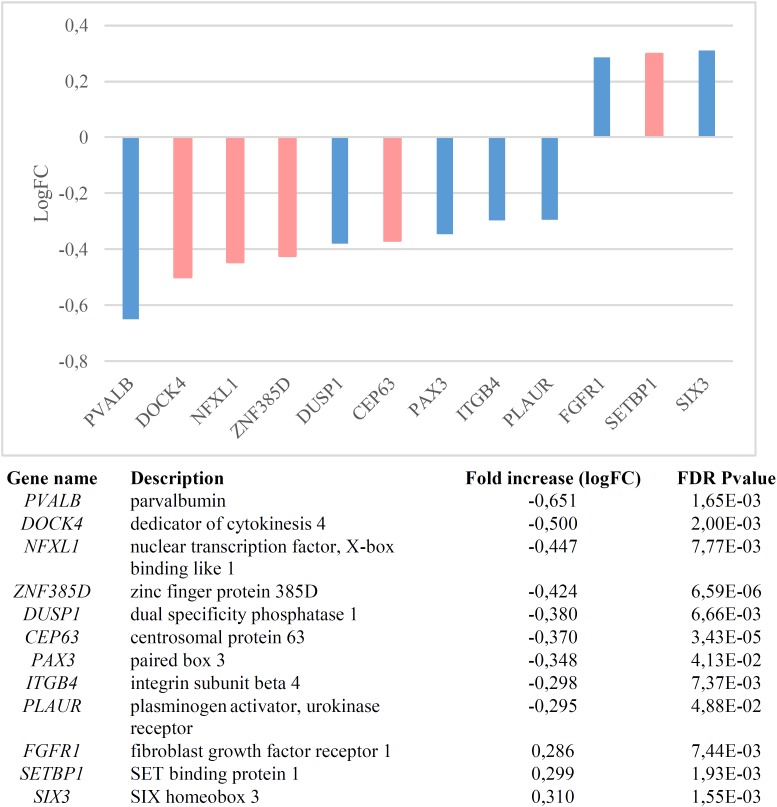
Genes significantly dysregulated in the blood of subjects with WS (FDR < 0.05, |FC| > 1.2). Candidate genes for language disorders (SLI, DD) are displayed in light red, whereas candidates for language evolution are colored in light blue.

We found that candidates for language (abnormal) development and language evolution are significantly dysregulated in the blood of subjects with WS (*p* = 1.1e-7 by Fisher’s exact test). Figure 1 shows the genes that are significantly up- or down-regulated compared to controls (FDR < 0.05, |FC| > 1.2).

In order to check the specificity of this set of genes in relation to language we conducted a functional enrichment analysis with Enrichr (amp.pharm.mssm.edu/Enrichr; Chen et al., 2013; Kuleshov et al., 2016), which showed that they are significantly related to biological processes, molecular functions, and abnormal phenotypes of interest for language (Table 1). Finally, these genes were predicted to be preferentially expressed in body parts important for language processing or for language development, particularly, the cerebellum and the thalamus (Table 1 and Supplementary Table S2). We now provide a detailed discussion of our results.

**Table 1:** Functional enrichment analysis according to Enrichr of the set of genes significantly dysregulated in the blood of subjects with WS.

Index	Name	*P*-value	Adjusted *p*-value	Combined *Z*-score	score
**GO Biological Process 2018**					
1	Regulation of endothelial cell chemotaxis to fibroblast growth factor (GO:2000544)	0.004791	0.04609	−3.72	19.88
2	Regulation of mitotic spindle checkpoint (GO:1903504)	0.004193	0.04609	−3.57	19.54
3	Positive regulation of vascular endothelial cell proliferation (GO: 1905564)	0.005985	0.04609	−3.47	17.74
4	Negative regulation of meiotic cell cycle (GO:0051447)	0.004193	0.04609	−3.21	17.58
5	Positive regulation of smooth muscle cell migration (GO:0014911)	0.006582	0.04609	−3.48	17.49
6	Embryonic camera-type eye morphogenesis (GO:0048596)	0.004193	0.04609	−2.98	16.34
7	Attachment of GPI anchor to protein (GO:0016255)	0.004791	0.04609	−2.95	15.76
8	Regulation of MAP kinase activity (GO:0043405)	0.001228	0.04609	−2.13	14.28
9	Regulation of neuroblast proliferation (GO: 1902692)	0.006582	0.04609	−2.83	14.20
10	Mesodermal cell differentiation (GO:0048333)	0.005985	0.04609	−2.71	13.87
**GO Molecular Function 2018**					
1	MAP kinase tyrosine/serine/threonine phosphatase activity (GO:0017017)	0.004791	0.07352	−3.07	16.41
2	MAP kinase phosphatase activity (GO:0033549)	0.005985	0.07352	−2.64	13.51
3	Transcription corepressor binding (GO:0001222)	0.007774	0.07352	−2.38	11.56
4	Insulin-like growth factor binding (GO:0005520)	0.008965	0.07352	−2.18	10.27
5	Insulin-like growth factor I binding (GO:0031994)	0.008370	0.07352	−2.10	10.02
6	Protein tyrosine kinase binding (GO: 1990782)	0.02901	0.08271	−2.70	9.55
7	Transcription cofactor binding (GO:0001221)	0.01549	0.08271	−2.29	9.54
8	Receptor tyrosine kinase binding (GO:0030971)	0.02374	0.08271	−2.35	8.80
9	Phosphatidylinositol kinase activity (GO:0052742)	0.03077	0.08271	−2.28	7.93
10	Fibroblast growth factor binding (GO:0017134)	0.01372	0.08271	−1.84	7.88
**GO Cellular Component 2018**					
1	Specific granule membrane (GO:0035579)	0.05384	0.2061	−1.87	5.47
2	Spindle pole (GO:0000922)	0.06293	0.2061	−1.79	4.95
3	Centriole (GO:0005814)	0.05612	0.2061	−1.53	4.40
4	Nucleolus (GO:0005730)	0.06032	0.2061	−1.54	4.33
5	Specific granule (GO:0042581)	0.09246	0.2133	−1.57	3.74
6	Microtubule organizing center part (GO:0044450)	0.07361	0.2061	−1.40	3.64
**MGI Mammalian Phenotype 2017**					
1	MP:0002151_abnormal_neural_tube_morphology	0.0001029	0.009725	−3.34	30.71
2	MP:0011089_perinatal_lethality,_complete_penetrance	0.0002578	0.01392	−3.68	30.40
3	MP:0012138_decreased_forebrain_size	0.00003942	0.007070	−2.67	27.06
4	MP:0002950_abnormal_neural_crest_cell_migration	0.00003942	0.007070	−2.48	25.10
5	MP:0010378_increased_respiratory_quotient	0.0002542	0.01392	−2.77	22.94
6	MP:0003864_abnormal_midbrain_development	0.0001725	0.01304	−2.45	21.19
7	MP:0000733_abnormal_muscle_development	0.00005611	0.007070	−2.11	20.66
8	MP:0005221_abnormal_rostral-caudal_axis_patterning	0.0005171	0.02443	−2.55	19.29
9	MP:0005602_decreased_angiogenesis	0.0006517	0.02463	−2.56	18.75
10	MP:0002111_abnormal_tail_morphology	0.0006517	0.02463	−2.47	18.08
**Human Phenotype Ontology**					
1	Malar flattening (HP:0000272)	0.00000345 5	0.0008259	−2.42	30.38
2	Midface retrusion (HP:0011800)	0.00000611 8	0.0008259	−2.02	24.26
3	Camptodactyly of finger (HP:0100490)	0.00004567	0.001761	−2.26	22.60
4	Choanal stenosis (HP:0000452)	0.00003450	0.001553	−2.15	22.14
5	Shallow orbits (HP:0000586)	0.00002171	0.001553	−2.02	21.67
6	Ureteral obstruction (HP:0006000)	0.00003450	0.001553	−2.08	21.39
7	Short nose (HP:0003196)	0.00007989	0.002546	−2.26	21.32
8	Ureteral stenosis (HP:0000071)	0.00002991	0.001553	−2.01	20.93
9	Heterogeneous (HP:0001425)	0.00008487	0.002546	−2.14	20.06
10	Depressed nasal bridge (HP:0005280)	0.0002981	0.007161	−2.37	19.20
**Jensen TISSUES**					
1	Ectoderm	0.00006469	0.005546	−4.15	39.98
2	Cranium	0.00007203	0.005546	−4.00	38.17
3	Retina	0.008991	0.09231	−7.36	34.68
4	Adult	0.01554	0.09772	−6.50	27.05
5	Neural crest	0.002981	0.06593	−4.14	24.10
6	Somite	0.0005354	0.02749	−3.08	23.19
7	Bud	0.001676	0.05163	−3.59	22.95
8	Mesenchyme	0.01050	0.09772	−5.01	22.84
9	Myoblast	0.001550	0.05163	−3.53	22.83
10	Immune system	0.02200	0.1019	−5.91	22.56

*Only the top-10 functions have been included (whenever available). The p-value was computed using Fisher’s exact test. The adjusted p-value was computed using the Benjamini-Hochberg method for correction for multiple hypotheses testing. The z-score was computed using a modification to Fisher’s exact test and assess the deviation from the expected rank. Finally, the combined score is a combination of the p-value and z-score calculated by multiplying the two scores (combined score = ln(p-value) ^∗^ z-score). This combined score provides a compromise between both methods and it is claimed to report the best rankings when compared with the other scoring schemes. See http://amp.pharm.mssm.edu/Enrichr/help#background&q=5 for details.*

## Discussion

### Functional Characterization of Individual Genes

Nearly one third of the language-related genes found downregulated in the blood of subjects with WS are candidates for DD (*DOCK4*, *ZNF385D*, and *CEP63*) and/or for SLI (*DOCK4*, *NFXL1*). As other members of the Dock family, DOCK4 regulates cytoskeleton assembly and cell adhesion and migration (Gadea and Blangy, 2014). Specifically, DOCK4 has been shown to be involved in neuronal migration and neurite differentiation (Ueda et al., 2008; Xiao et al., 2013), via interaction with the actin-binding protein cortactin (Ueda et al., 2013). Knockdown of *Dock4* in mice abolishes commissural axon attraction by Shh (Makihara et al., 2018). The gene has been related to neuronal migration and neurite outgrowth anomalies linked to DD (Shao et al., 2016), although it is also associated with ASD (Pagnamenta et al., 2010) and SZ (Alkelai et al., 2012). GWAs have associated markers in *ZNF385D* to the co-occurrence of reading disability and language impairment (Eicher et al., 2013), but also to negative symptoms in SZ (Xu et al., 2013). *CEP63* is required for normal spindle assembly, being involved in maintaining centriole number and establishing the order of events in centriole formation (Brown et al., 2013). Besides its association with DD (Einarsdottir et al., 2015), the gene is also a candidate for primary microcephaly (Marjanović et al., 2015), a feature that is commonly found in subjects with WS (Jernigan and Bellugi, 1990; Schmitt et al., 2001; Thompson et al., 2005; Jackowski et al., 2009). Finally, variants of *NFXL1*, which is predicted to encode a transcription factor, confer a risk for SLI (Villanueva et al., 2015). The gene is highly expressed in the cerebellum (Nudel, 2016).

Regarding the candidates for language evolution that we have found downregulated in the blood of subjects with WS, *DUSP1* is involved in vocal learning in songbirds (Horita et al., 2010, 2012). *PVALB* encodes a calcium-binding protein that is structurally and functionally similar to calmodulin and that is involved in hippocampal plasticity, learning and memory (Donato et al., 2013). Interestingly enough, the inactivation of Pvalb-expressing interneurons in the auditory cortex alters response to sound, strengthening forward suppression and altering its frequency dependence (Phillips et al., 2017). Inhibition of PVALB-expressing GABAergic interneurons results in complex behavioral changes related to the behavioral phenotype of people with SZ (Brown et al., 2015). Importantly, some of the key changes that contributed to the emergence of our language-readiness involved GABAergic signaling (discussed in detail in Boeckx and Benítez-Burraco, 2014b), which are vital for oscillatory processes underlying language processing (Bae et al., 2010; see Murphy and Benítez-Burraco, 2018 for details). Reduction in *PVALB* expression in interneurons has also been found in mouse models of ASD (Filice et al., 2016), specifically, in the Cntnap2-/- model (Lauber et al., 2018). *CNTNAP2* is a direct target of FOXP2, the renowned “language gene” (Vernes et al., 2008; Adam et al., 2017), and regulates language development in non-pathological populations too (Whitehouse et al., 2011; Whalley et al., 2011, Kos et al., 2012). Also mice lacking *PLAUR* have significantly fewer neocortical parvalbumin-containing GABAergic interneurons, with this reduction correlating with impaired social interactions (Bruneau and Szepetowski, 2011). *PLAUR* is a target of FOXP2 too (Roll et al., 2010), but also an effector of SRPX2, another of FOXP2’s targets (Royer-Zemmour et al., 2008) and a candidate for speech dyspraxia (Roll et al., 2006). Concerning PAX3, this gene is expressed in the neural crest and is a candidate for Waardenburg syndrome, a clinical condition entailing sensorineural hearing loss and developmental delay (Tassabehji et al., 1992; Chen et al., 2010). Finally, *ITGB4* encodes the integrin beta 4 subunit, a receptor for the laminins, including FLNA (Travis et al., 2004), an actin-binding protein needed for cytoskeleton remodeling and neuronal migration (Fox et al., 1998) FLNA binds *CMIP* (Fox et al., 1998), a candidate for SLI (Newbury et al., 2009). Interestingly enough, ITGB4 is one of the proteins bearing fixed changes in humans compared to extinct hominins (Pääbo, 2014; Supplementary Table S1).

Lastly, among the genes found to be upregulated in the blood on WS subjects, we found the SLI candidate *SETBP1*, as well as *FGFR1* and *SIX3*. SETBP1 is also a candidate for Schinzel-Giedion syndrome, a clinical condition entailing occasional epilepsy and severe developmental delay (Ko et al., 2013; Miyake et al., 2015). Mutations on this gene have been associated as well to behavioral and social deficits (Coe et al., 2014). The Integrative Nuclear FGFR1 Signaling (INFS) has been hypothesized to be one of the neurodevelopmental pathways on which multiple SZ candidates converge, regulating numerous neurotransmitter systems and neural circuits (Stachowiak et al., 2013). Finally, *SIX3* contributes to regulate the relative size of the telencephalon versus the thalamus (Lavado et al., 2008; Sylvester et al., 2010). Interestingly, Six3 regulates Shh (Jeong et al., 2008), one robust candidate for microcephaly that has been positively selected in the human lineage (Dorus et al., 2004), but it also interacts with several genes relevant for our language-ready brain (Benítez-Burraco and Boeckx, 2015).

### Functional Characterization of the Set of Dysregulated Genes

The results of our functional enrichment analyses (Table 1) show that the language-related genes that are dysregulated in the blood of people with WS mainly contribute to the cytoskeleton activity, being significantly involved in cell proliferation and migration, including neuroblast proliferation. Regarding their molecular function, they typically participate in protein modification, particularly via (tyrosine) kinase phosphatase and (tyrosine) kinase binding activities, but also in gene regulation, via transcription cofactor binding. Interestingly, these genes are significantly associated to aberrant processes impacting on brain development, like abnormal neural tube morphology and neural crest cell migration, as well as decreased forebrain size and abnormal midbrain development. Likewise, they are associated to clinical symptoms mostly impacting on craniofacial morphology, like malar flattening, midface retrusion, shallow orbits, or depressed nasal bridge. Finally, these genes are predicted to be preferentially expressed in the ectoderm, the cranium, the retina, and the neural crest. According to the Human Brain Transcriptome Database^1^ all these genes are expressed in the brain, particularly in the thalamus and the cerebellum (Supplementary Table S2). The thalamus functions as a sort of relay center to connect many brain areas involved in language processing (Wahl et al., 2008; Murdoch, 2010; David et al., 2011) and changes in the thalamus have been claimed to contribute to the evolutionary emergence of our language-ready brain (see Boeckx and Benítez-Burraco, 2014b for details). Similarly, the cerebellum plays a key role in language processing and is impaired in language-related pathologies (Vias and Dick, 2017; Mariën and Borgatti, 2018). People with WS exhibit cerebellar volume alterations that are seemingly associated with their cognitive, affective and motor distinctive features (Osório et al., 2014). In the same vein, the thalamus exhibits structural and functional differences with the neurotypical population, including disproportionately reduced volumes and decreased gray matter (Chiang et al., 2007; Campbell et al., 2009), as well as enhanced thalamic activity (Mobbs et al., 2007; Bódizs et al., 2012).

## Conclusion

To conclude, it is true that deciphering the exact molecular causes of language dysfunction in WS is still pending, particularly, because at present none of the genes hemideleted in this condition has been demonstrated to play a central role in language processing. Nonetheless, in this paper we have shown that the genes that are dysregulated in subjects with WS are significantly enriched in core candidates for language disorders and language evolution. These genes emerge as robust candidates for language dysfunction in WS. Future research should try to delve into the expression patterns of these genes in the brain of people with WS, as well as into their role in neurotypical brain development. Likewise, altering these genes in animal models of WS should help gaining a better understanding of their biological role and ultimately, of their contribution to language dysfunction in WS.

## Data Availability

Publicly available datasets were analyzed in this study. This data can be found here: https://www.ncbi.nlm.nih.gov/geo/query/acc.cgi?acc=GSE89594.

## Author Contributions

AB-B conceived and wrote the manuscript. RK conducted the expression studies and analyzed the data. Both authors contributed to manuscript revision, and read and approved the submitted version.

## Conflict of Interest Statement

The authors declare that the research was conducted in the absence of any commercial or financial relationships that could be construed as a potential conflict of interest.

## Footnotes

1 http://hbatlas.org
